# Interlayer Separation in Graphene Paper Comprising Electrochemically Exfoliated Graphene

**DOI:** 10.3390/nano11040865

**Published:** 2021-03-29

**Authors:** Dang Du Nguyen, TaeGyeong Lim, Soomook Lim, Ji Won Suk

**Affiliations:** 1 School of Mechanical Engineering, Sungkyunkwan University, Suwon 16419, Gyeonggi-do, Korea; dangdunguyen.bku@gmail.com (D.D.N.); taegyung95@gmail.com (T.L.); growing18@naver.com (S.L.); 2 Department of Smart Fab. Technology, Sungkyunkwan University, Suwon 16419, Gyeonggi-do, Korea; 3 SKKU Advanced Institute of Nanotechnology (SAINT), Sungkyunkwan University, Suwon 16419, Gyeonggi-do, Korea

**Keywords:** graphene paper, electrochemical exfoliation, interlayer separation, fracture, double cantilever beam

## Abstract

The emergence of graphene paper comprising well-stacked graphene flakes has promoted the application of graphene-based materials in diverse fields such as energy storage devices, membrane desalination, and actuators. The fundamental properties of graphene paper such as mechanical, electrical, and thermal properties are critical to the design and fabrication of paper-based devices. In this study, the interlayer interactions in graphene paper were investigated by double cantilever beam (DCB) fracture tests. Graphene papers fabricated by flow-directed stacking of electrochemically exfoliated few-layer graphene flakes were mechanically separated into two parts, which generated force-displacement responses of the DCB sample. The analysis based on fracture mechanics revealed that the interlayer separation energy of the graphene paper was 9.83 ± 0.06 J/m^2^. The results provided a fundamental understanding of the interfacial properties of graphene papers, which will be useful for developing paper-based devices with mechanical integrity.

## 1. Introduction

Graphene has attracted considerable interest from researchers in academia and industry owing to its exceptional mechanical, electrical, optical, and thermal properties [1,2,3,4,5]. These properties facilitate the utilization of graphene in various advanced applications, such as energy storage devices, nanoelectronics, optoelectronics, nanocomposites, and flexible devices [6,7,8,9,10,11,12]. High-quality and stable single-layer graphene on SiO_2_ can be obtained via the mechanical exfoliation of bulk graphite using scotch tape [1]. However, this technique is not suitable for mass production due to various issues such as low lateral sizes and random positions of the graphene flakes; in addition, it is difficult to control the number of layers of the graphene flakes. Therefore, several methods have been developed for the production of graphene in substantial quantities, such as epitaxial growth on SiC [13], chemical vapor deposition (CVD) on metal substrates [14], and oxidation of graphite followed by reduction of graphene oxide (GO) [15]. The oxidation of graphite, subsequent exfoliation of graphite oxide to GO, and eventual chemical or thermal reduction of GO is a scalable and inexpensive technique to obtain substantial quantities of graphene flakes [16]. GO and reduced graphene oxide (rGO) have been extensively utilized as components of paper-like materials [17,18], nanocomposites [15,19], electrodes for energy storage devices [20,21], transparent conducting films [22], and high-performance coatings [23]. However, the oxidation of graphite induces the formation of numerous defects and oxygen functional groups [24]. The direct exfoliation of graphite using electrochemical exfoliation, without the involvement of hazardous chemicals, is a viable alternative to the use of colloidal suspensions of GO [25,26]. Electrochemically exfoliated graphene (EEG) exhibits a high C/O atomic ratio and minimal defects. Therefore, it has been extensively utilized in various applications, such as electrodes of supercapacitors and batteries, field-effect transistors, conductive fillers in polymer composites, and conductive inks [25,26,27,28,29,30,31,32,33].

Macroscopic paper-like materials, among the multiple applications of graphene-based materials, exhibit high potential for utilization in free-standing, lightweight, and flexible devices, such as energy storage devices, membranes, sensors, and actuators [34,35]. Mechanical integrity is a fundamental property of graphene papers, and it is crucial for the development of reliable and high-performance paper-based devices. Graphene papers are formed by layer-by-layer assembly; therefore, the interlayer interaction significantly influences the mechanical properties of these papers. There has been extensive research on strengthening the interactions between the graphene layers to increase the fracture strengths and Young’s moduli of graphene papers. It has been reported that the mechanical properties of GO papers can be enhanced by chemical cross-linking using divalent metal ions [36]. A decrease in the interlayer spacing of EEG paper strengthens the interlayer interactions, thereby increasing the fracture strength of graphene papers [37]. However, there is limited knowledge about the interlayer interactions in graphene papers.

In this work, the interlayer separation in graphene paper was investigated using mode I fracture tests. Graphene flakes were obtained using electrochemical exfoliation of graphite; subsequently, graphene papers were fabricated by flow-directed assembly of the obtained graphene flakes. The double cantilever beam (DCB) specimens for the mode I fracture tests were prepared by gluing both sides of the graphene paper to two Si substrates. The interlayer separation energy was estimated using the force–displacement responses obtained from the mechanical separation of the graphene paper. 

## 2. Materials and Methods

### 2.1. Fabrication of Graphene Papers Using EEG Flakes 

Graphene flakes were synthesized by the electrochemical exfoliation of graphite (Supplementary Figure S1a) [38]. A commercial graphite foil (99.8% purity (metal basis); Alfa Aesar, Haverhill, MA, USA) with a thickness of 0.5 mm was cut into 5 cm-long and 1 cm-wide strips for application as a working electrode (Supplementary Figure S2a). A Pt plate was utilized as a counter electrode and placed parallel to the graphite electrode at a fixed distance of 2 cm [39]. A 0.1 M electrolyte solution was prepared by the dissolution of ammonium sulfate ((NH_4_)_2_SO_4_; Sigma-Aldrich, St. Louis, MO, USA) in water and subsequent stirring at 100 rpm for 10 min. Electrochemical exfoliation was initiated by applying a DC voltage (2 V) to the graphite for 3 min. This facilitated wetting of the graphite electrode and intercalation of the electrolyte anions that induced lattice expansion in graphite. Exfoliation was completed under an applied voltage of 10 V. Thereafter, the suspended graphene sheets were collected using vacuum filtration through a polytetrafluoroethylene (PTFE) membrane filter (pore size = 0.2 µm) and washed several times with ethanol and deionized water (Supplementary Figure S1b). This was followed by drying in an oven for 12 h at 60 °C to remove the moisture in the powders. The dried graphene powders were dispersed in dimethylformamide (DMF) by sonication for 30 min. The EEG suspension was subjected to centrifugation at 3000 rpm for 30 min to remove the thick graphene flakes. Graphene papers were fabricated by vacuum filtration of the EEG dispersion (0.15 mg/mL in DMF, Supplementary Figure S2b) using an anodic aluminum oxide (AAO) membrane filter (pore size = 0.2 µm; Whatman, Maidstone, UK). 

### 2.2. Characterization of EEG Paper

The EEG papers were subjected to morphological analysis using scanning electron microscopy (SEM; JSM-7600, Jeol Ltd., Tokyo, Japan). The EEG flakes were placed on a SiO_2_/Si substrate; thereafter, their lateral sizes and thicknesses were determined using SEM and atomic force microscopy (AFM; 5300E, Hitachi High-Technologies Corporation, Tokyo, Japan), respectively. The EEG papers and their fracture surfaces were characterized by Raman spectroscopy (alpha300M, WITec GmbH, Ulm, Germany) with a laser wavelength of 532 nm. The chemical structures of the EEG papers were characterized using Fourier-transform infrared (FTIR) spectroscopy (IFS-66/S, Tensor 27, Bruker Optik GmbH, Ettlingen, Germany) and X-ray photoelectron spectroscopy (XPS; Escalab-250 (with monochromated Al Kα radiation), Thermo Fisher Scientific, Waltham, MA, USA). The C 1s peak of the XPS spectra was deconvoluted using Gaussian–Lorentzian peak shapes after Shirley background correction [40]. The crystalline structures of the EEG papers were characterized by X-ray diffraction (XRD; D8 Advance, Bruker AXS GmbH, Karlsruhe, Germany) using Cu Kα radiation. Thermogravimetric analysis (TGA; EXSTAR TG/DTA6100, Seiko Instruments Inc., Chiba, Japan) was performed at a temperature range of 25–600 °C under nitrogen atmosphere. The electrical conductivity of the EEG paper was measured by the van der Pauw method with four-point probes.

### 2.3. Interlayer Separation in EEG Paper

The DCB specimens (5 × 30 mm) were prepared by sandwiching the EEG paper between two Si substrates using a high-viscosity adhesive (302/8OZ, Epoxy Technology, MA, USA). The prepared DCB specimens exhibited a configuration of Si/adhesive/graphene paper/adhesive/Si (Supplementary Figure S3a). The laminated samples were cured at 100 °C for 2 h, and Al loading tabs were attached to one side of each Si substrate for the application of a tensile force. 

Glue was not applied at one end of the substrate, where the loading tabs were attached, to allow the formation of an initial crack. The initial crack length (*a_o_*) was determined from the distance between the center point of the loading tab and the crack front. The DCB specimens were mechanically separated by mode I fracture tests at a constant displacement rate of 0.5 mm/min using a universal testing machine (QC-506M2, Cometech, Taiwan) (Supplementary Figure S3b). The crack length (*a*) at any point during the DCB fracture test was estimated from the force–displacement responses using simple beam theory, according to the following equation [41,42]:

(1) $$a^{3}=Ebh^{3}\Delta /8P,$$

where *P* and Δ represent the load and applied displacement, respectively; furthermore, *E*, *b*, and *h* denote the in-plane Young’s modulus, width, and thickness of the substrate, respectively. The interlayer separation energy of the graphene paper was determined from the energy release rate (*G*) obtained using the following equation [41,42]:

(2) $$G=12a^{2}P^{2}/Eh^{3}b^{2},$$

## 3. Results and Discussion

### 3.1. Characterization of EEG Papers

**Figure 1:**
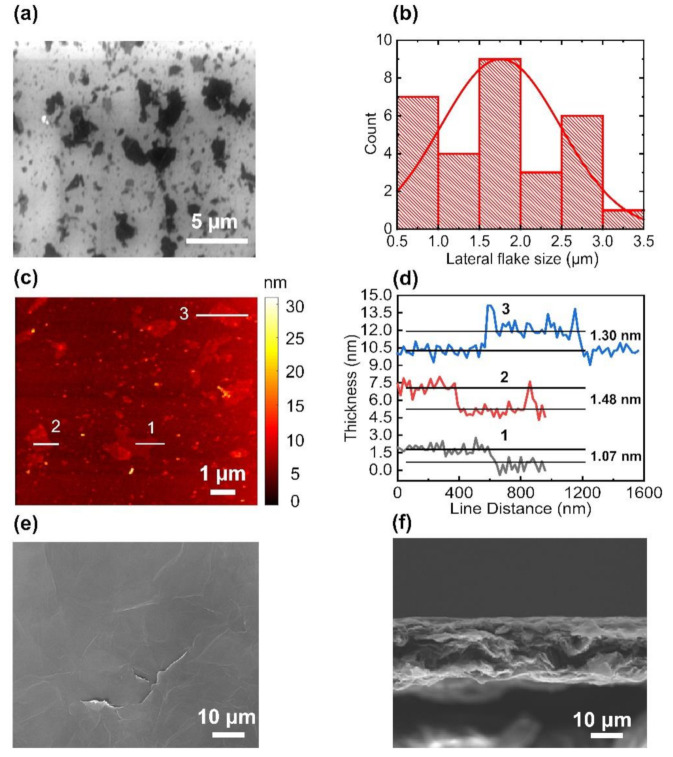
Morphologies of the EEG flakes and papers: (**a**) SEM image of the EEG flakes placed on an SiO_2_/Si substrate; (**b**) histogram of the lateral sizes of the EEG flakes, estimated from the SEM image (the red solid lines represent the Gaussian fits to the data); (**c**) AFM image of the EEG flakes placed on an SiO_2_/Si substrate; (**d**) representative AFM line profiles of the EEG flakes; (**e**) SEM image of the surface morphology of the EEG paper; (**f**) cross-sectional SEM image of the EEG paper.

The EEG flakes were dispersed on a SiO_2_/Si substrate, and their lateral sizes were estimated from the SEM images (Figure 1a). Most of the flakes exhibited sizes of less than approximately 3 µm (Figure 1b). The AFM observations revealed that the EEG flakes comprised a few layers (Figure 1c). The thickness of the EEG flakes was determined to be approximately 1–1.5 nm based on the line profiles presented in Figure 1d. These results indicated that the morphologies of the EEG flakes in this study were similar to those of the previously reported EEG flakes on an SiO_2_ substrate [32]. The EEG paper, obtained by vacuum filtration, exhibited uniform and smooth surfaces with negligible wrinkles (Figure 1e). The cross-sectional SEM image in Figure 1f confirmed the layer-by-layer stacking of the EEG flakes in the paper (thickness = 19.6 ± 1.4 µm).

**Figure 2:**
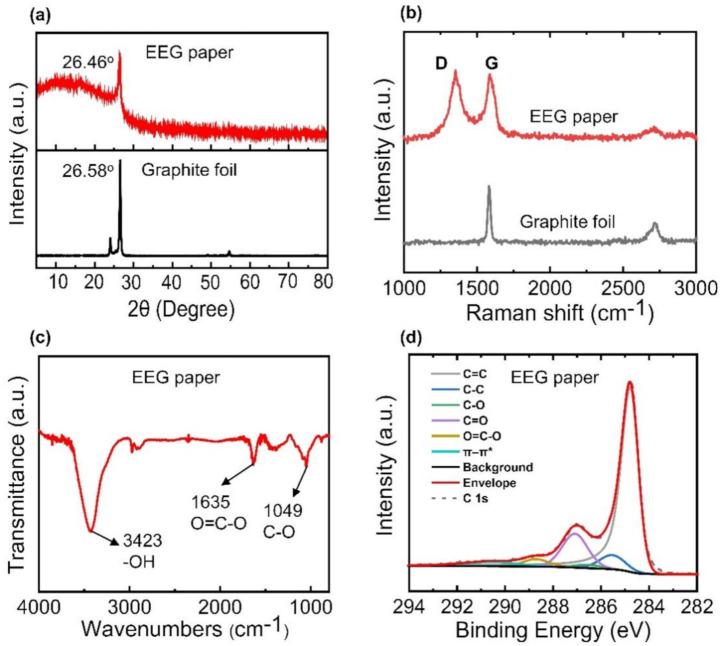
(**a**) XRD patterns and (**b**) Raman spectra of a graphite foil and the EEG paper; (**c**) FTIR spectrum and (**d**) XPS C 1s core-level spectrum of the EEG paper.

The interlayer structure of the EEG paper was characterized by thin-film XRD analysis. The XRD pattern of the EEG paper exhibited a prominent peak at 26.46° corresponding to a d-spacing of 0.3364 nm (Figure 2a). The XRD pattern of a graphite foil is also presented in Figure 2a, and it exhibited a peak at 26.58° corresponding to a d-spacing of 0.3349 nm. The slightly higher d-spacing of the EEG paper than that of the graphite foil was attributed to the presence of oxygen functional groups generated by electrochemical exfoliation [25,32]. The Raman spectrum of the EEG paper depicted the characteristic features of the graphene flakes (Figure 2b). The D band, G band, and 2D band were observed at approximately 1352, 1584, and 2680 cm^−1^, respectively. The D band corresponded to defects such as functional groups or structural disorders in the sp^2^ carbon network of the EEG flakes. The peak intensity ratio of the D band to the G band, i.e., I_D_/I_G_, for the EEG paper (1.08) was similar to that for chemically or thermally reduced GO flakes (~1.2–1.5) [43,44].

The results of FTIR spectroscopy indicated the presence of oxygen functional groups in the EEG flakes (Figure 2c). The characteristic peaks in the FTIR spectrum at 3423, 1635, and 1049 cm^−1^ corresponded to the hydroxyl, carboxyl, and alkoxy groups, respectively [45,46,47]. The chemical structure of the EEG paper was investigated by XPS. The C 1s spectrum was fitted with six spectral components corresponding to C=C, C-C, C-O, C=O, O=C-O, and a π-π* shake-up at 284.8, 285.5, 286.5, 287.1, 288.7, and 290.5 eV, respectively [39,48,49,50,51]. The C/O atomic ratio of EEG (~3.4), obtained by XPS analysis, was higher than that of GO [52,53,54]. However, the C/O ratio was relatively lower than those of other graphene flakes synthesized by the electrochemical exfoliation method, which might be attributed to more oxidation during the exfoliation process [55]. Moreover, TGA on the EEG paper showed the weight loss associated with the thermal removal of the oxygen functional groups in the paper (Supplementary Figure S4) [55]. In addition, the EEG paper exhibited a relatively lower electrical conductivity of 2570 ± 980 S/m compared to those of other graphene papers, which is consistent with the lower C/O ratio of the EEG paper [56].

### 3.2. Interlayer Separation in the EEG Paper Using Mode I Fracture

**Figure 3:**
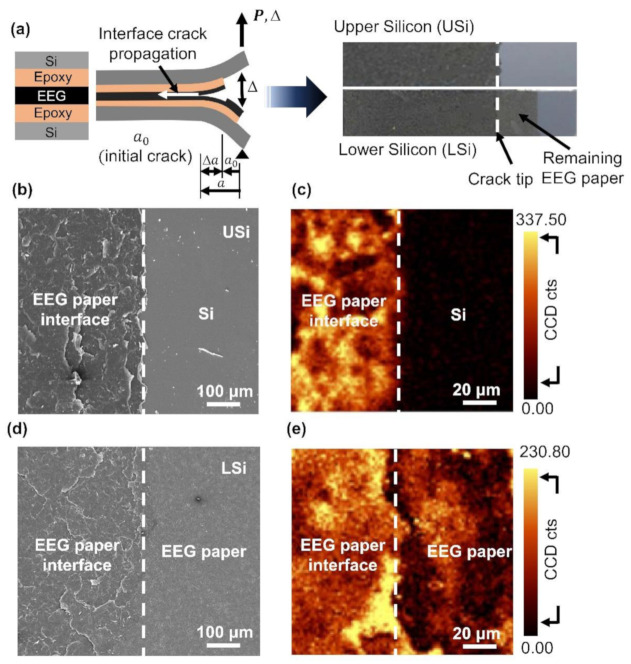
Characterization of the fracture surface of the EEG paper after the mode I fracture test: (**a**) schematic illustration of the interlayer separation in the EEG paper and a photograph of the DCB specimen after fracture; (**b**,**d**) SEM images and (**c**,**e**) Raman intensity maps of the D band for the fracture surfaces of the USi and LSi near the epoxy terminus.

The EEG paper was subjected to mode I fracture to elucidate the interlayer interactions between the graphene flakes. The photographs of the upper and lower Si substrates (USi and LSi) after the fracture showed the uniform dark color of the entire substrate (Figure 3a). This indicated the complete coverage of the EEG flakes over the adhesive on the Si substrates. The SEM image of the USi revealed the presence of the EEG paper in the epoxy region (Figure 3b). The complementary fracture surface of the EEG paper was observed in the left region of the LSi, while the original EEG paper was observed in the right region of the LSi (Figure 3d). This was attributed to the absence of epoxy in the right region of the USi. The Raman intensity maps of the D band (1310–1390 cm^−1^) revealed the presence of EEG flakes near the epoxy terminus (Figure 3c,e). The entire fracture surfaces of the USi and LSi were subjected to SEM observations and Raman mapping. The results confirmed the uniform separation of the EEG paper into the USi and LSi (Supplementary Figures S5–S7).

**Figure 4:**
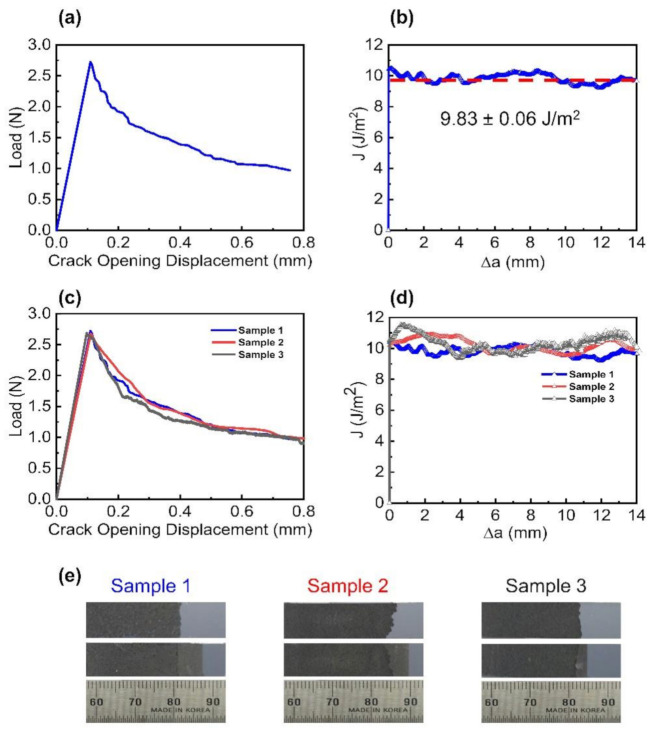
Interlayer separation behavior of the EEG paper: (**a**) force–displacement responses and (**b**) corresponding fracture resistance curve; (**c**) force–displacement curves and (**d**) corresponding fracture resistance curves of the three DCB specimens. Interfacial fracture behavior of the interface of the EEG paper. The red dashed line in (**b**) represents the average interlayer separation energy obtained from (**d**); (**e**) photograph of the fracture surfaces of the three DCB specimens.

Figure 4a presents the force–displacement responses for the interlayer separation in the EEG paper. When the specimen was subjected to monotonic loading, there was a linear increase in the force before the occurrence of fracture. A sudden decrease in the force indicated crack initiation corresponding to the interlayer separation in the EEG paper. There was an overall decrease in the force owing to crack propagation. The similar force–displacement curves and uniform fracture surfaces of the three specimens indicated the excellent repeatability of the measurements (Figure 4c,e). The interlayer separation energy of the EEG paper was estimated from the force–displacement responses based on the concept of linear elastic fracture mechanics. The J-integral (*J*), determined from the energy release rate (*G*), represented the interlayer bonding energy of the EEG paper (Figure 4b). The interlayer separation energy of the three specimens was determined to be 9.83 ± 0.06 J/m^2^ (Figure 4d), as depicted by the red dashed line in Figure 4b. The interlayer separation energy of the EEG paper was higher than the adhesion energies for previously reported graphene-based systems. The adhesion energy between monolayer graphene, obtained by mechanical exfoliation, and an SiO_2_ substrate was reported to be 0.45 J/m^2^ [57]. The adhesion energy between monolayer graphene, synthesized by CVD, and a seed Cu foil was reported to be 6 J/m^2^ [41], while that between graphene and highly oriented pyrolytic graphite was reported to be 0.088 J/m^2^ [58]. The adhesion energy between graphite and a diamond tip was reported to be 0.0259–0.1871 J/m^2^ [59]. The high interlayer separation energy of the EEG paper was attributed to the oxygen functional groups in the EEG flakes that induced strong layer-to-layer interactions [60]. The layer-by-layer assembly of the EEG paper resulted in a microstructure comprising interlocked EEG flakes, and this also contributed to the high interfacial cohesion energy [37]. Therefore, the energy required for interlayer separation was higher for the EEG paper than that for other two-dimensional materials [61,62].

## 4. Conclusions

Mechanical integrity of graphene papers is important for developing reliable paper-based devices. In this study, a classical fracture test was performed to induce interlayer separation in graphene paper, and the interlayer interactions between the graphene flakes were elucidated. Graphene flakes were obtained by electrochemical exfoliation of graphite; thereafter, graphene papers were fabricated via vacuum filtration of the obtained graphene flakes. The mode I fracture tests successfully separated the EEG paper into two parts; SEM and Raman spectroscopy observation on the fracture surfaces confirmed a complete separation of the paper. Therefore, the force–displacement responses obtained from the fracture tests revealed the interlayer separation energy in the EEG paper of 9.83 ± 0.06 J/m^2^. Since the EEG flakes were oxidized during the exfoliation process, the high separation energy was attributed to the strong interactions induced by the oxygen functional groups in the flakes. Furthermore, the interlocking of the individual EEG flakes in the paper might be another reason for the high separation energy. A better understanding of the interlayer interactions in graphene paper will facilitate the development of paper-based devices. 

## Data Availability

Not applicable.

## Supplementary Materials

The followings are available online at https://www.mdpi.com/article/10.3390/nano11040865/s1, Figure S1: Schematic illustration of the fabrication of the EEG paper; Figure S2: Photograph of (a) graphite foils and (b) dispersed EEG flakes in DMF; Figure S3: Schematic illustration of the mode I fracture tests of the EEG paper; Figure S4: TGA curve of the EEG paper; Figure S5: SEM images of the fracture surfaces of the upper and lower Si strips after the fracture tests; Figure S6: Raman spectra of the EEG paper after fracture; Figure S7: Raman intensity maps of the D band and G band for the fracture surface.
