# Supplementary material for: Interlayer Separation in Graphene Paper Comprising Electrochemically Exfoliated Graphene
Source: Nanomaterials (Basel). 2021 Mar 29;11(4):865. doi: 10.3390/nano11040865 (PMC8066209; doi:10.3390/nano11040865)
Supplement: Supplementary file 1 [file nanomaterials-11-00865-s001.pdf]

Supplementary Materials

Interlayer Separation in Graphene Paper Comprising Electrochemically Exfoliated Graphene

Dang Du Nguyen [1], TaeGyeong Lim [1], Soomook Lim [1] and Ji Won Suk [1,2,3,*]

[1] School of Mechanical Engineering, Sungkyunkwan University, Suwon, Gyeonggi-do 16419, Korea; dangdunguyen.bku@gmail.com (D.D.N.); taegyung95@gmail.com (T.L.); growing18@naver.com (S.L.)
[2] Department of Smart Fab. Technology, Sungkyunkwan University, Suwon, Gyeonggi-do 16419, Korea
[3] SKKU Advanced Institute of Nanotechnology (SAINT), Sungkyunkwan University, Suwon, Gyeonggi-do 16419, Korea
* Correspondence: jwsuk@skku.edu

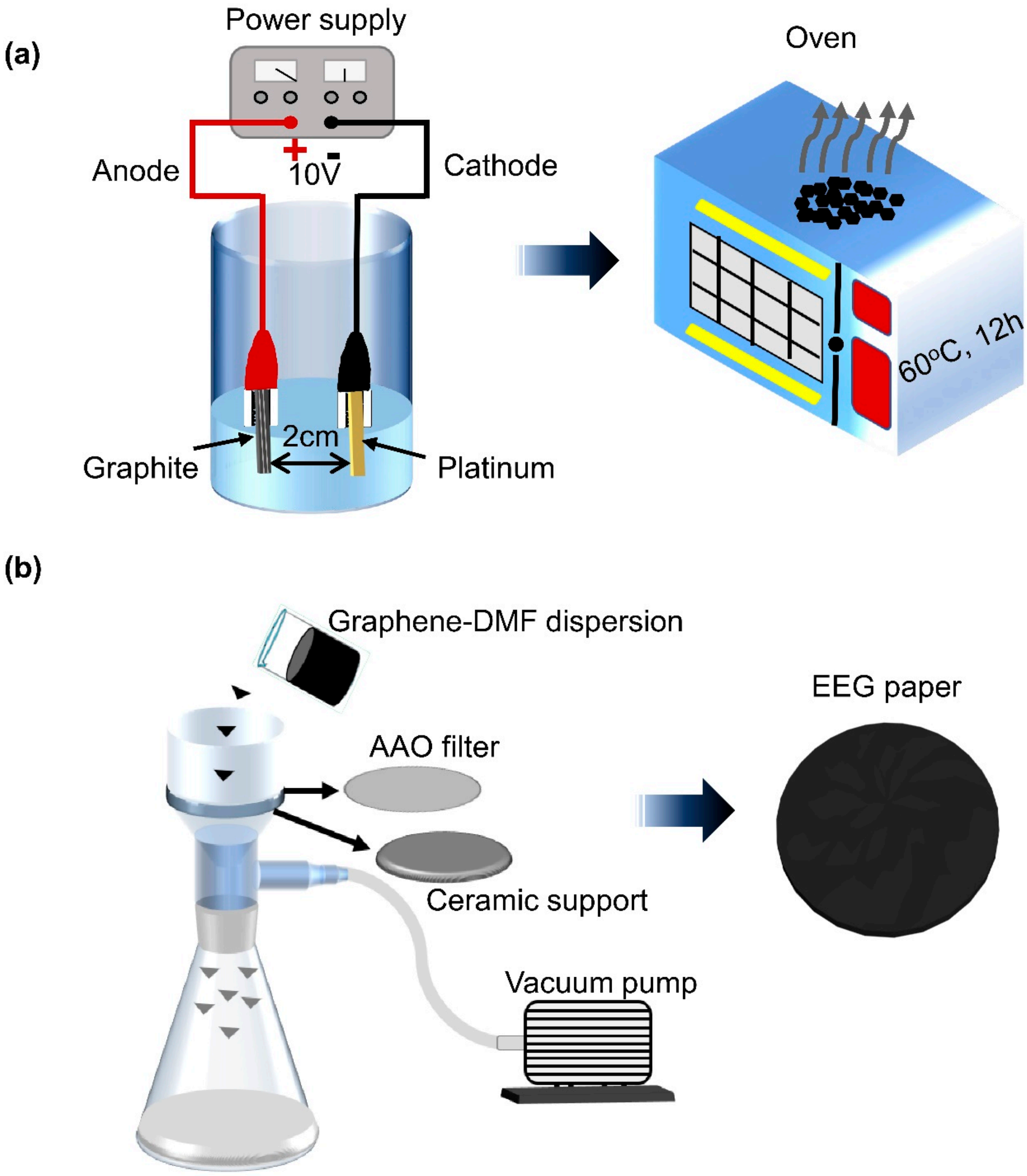

Figure S1. Schematic illustration of the fabrication of the EEG paper: (a) Electrochemical exfoliation of graphite. (b) Preparation of the EEG paper using vacuum filtration.

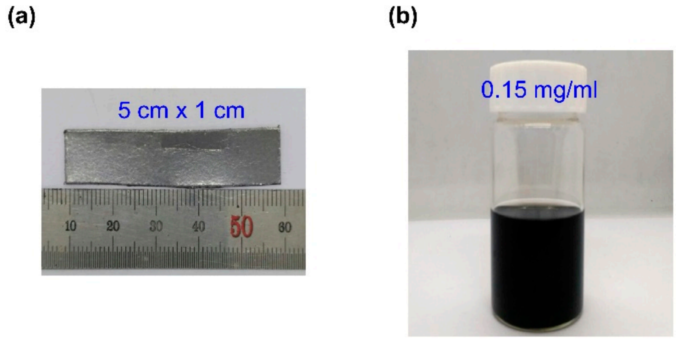

Figure S2. Photograph of (a) graphite foils and (b) dispersed EEG flakes in DMF.

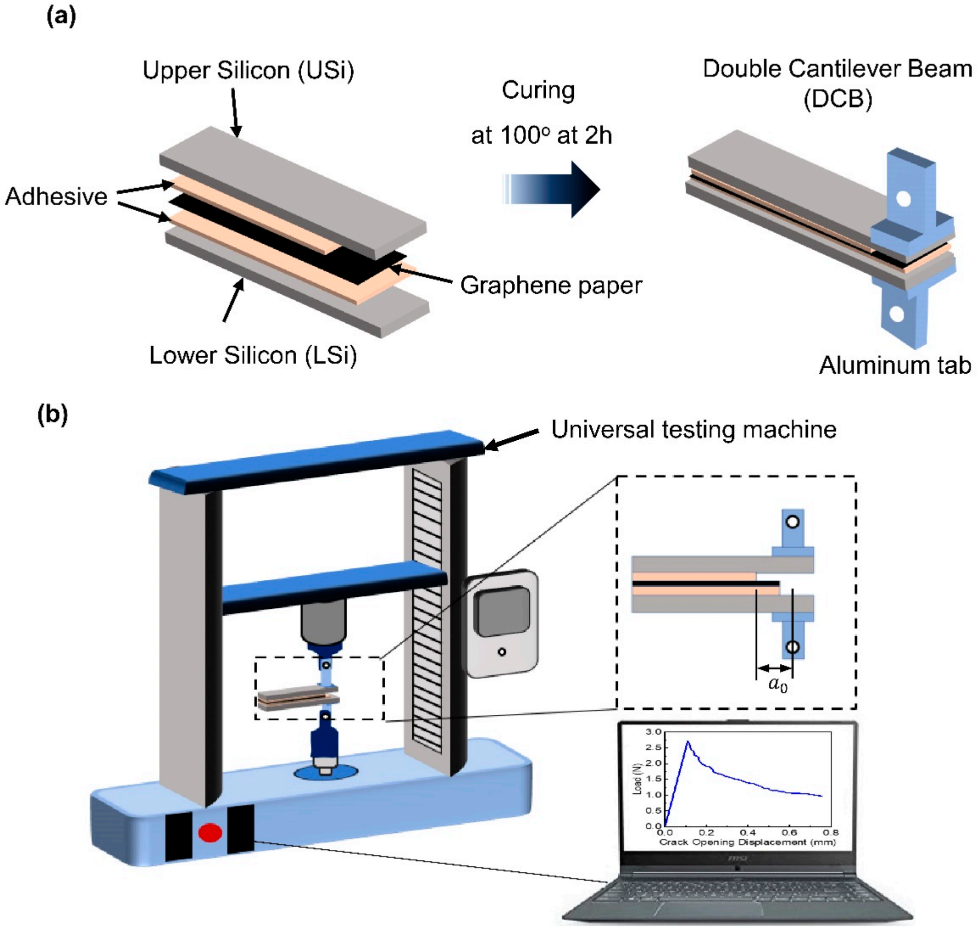

Figure S3. Schematic illustration of the mode I fracture tests of the EEG paper: (a) Preparation of the DCB specimen using the EEG paper. (b) Mechanical separation of the EEG paper using a universal testing machine.

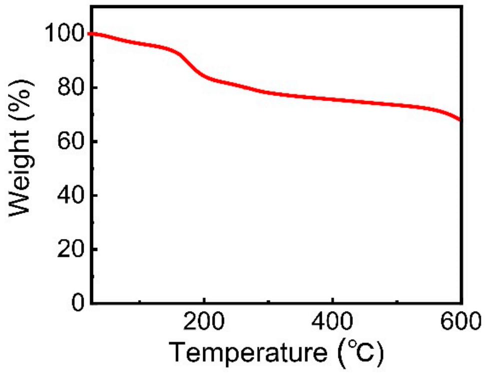

Figure S4. TGA curve of the EEG paper.

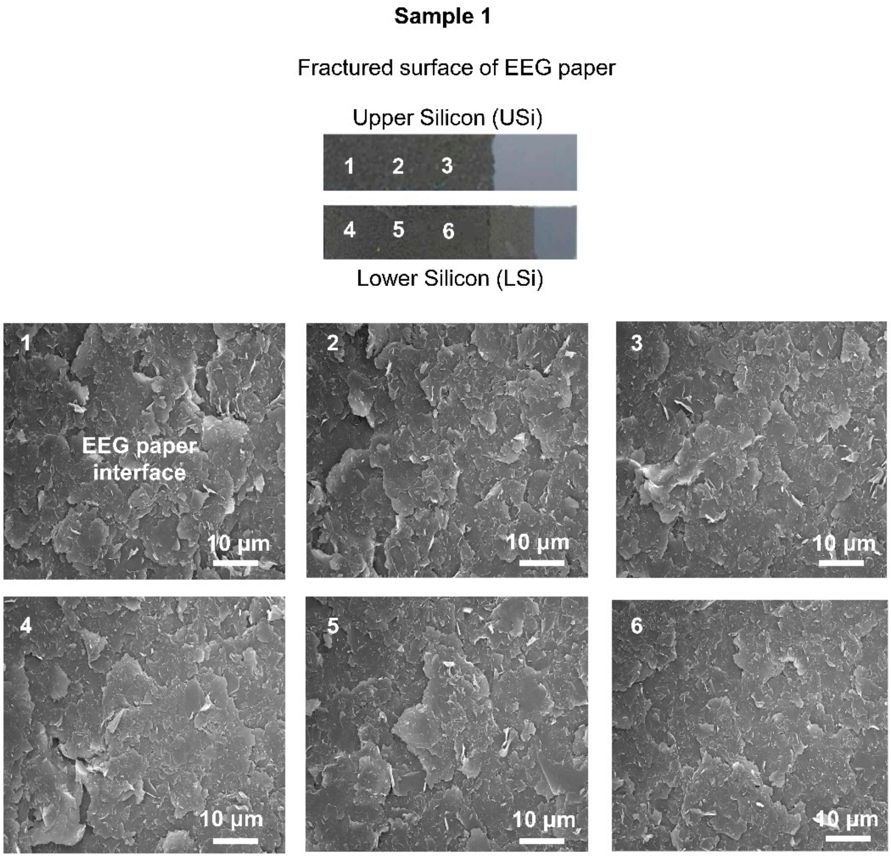

Figure S5. SEM images of the fracture surfaces of the upper and lower Si strips after the fracture tests.

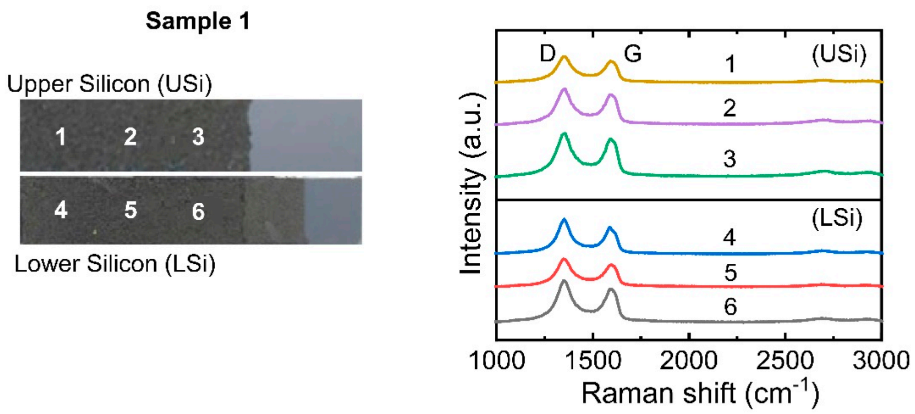

Figure S6. Raman spectra of the EEG paper after fracture.

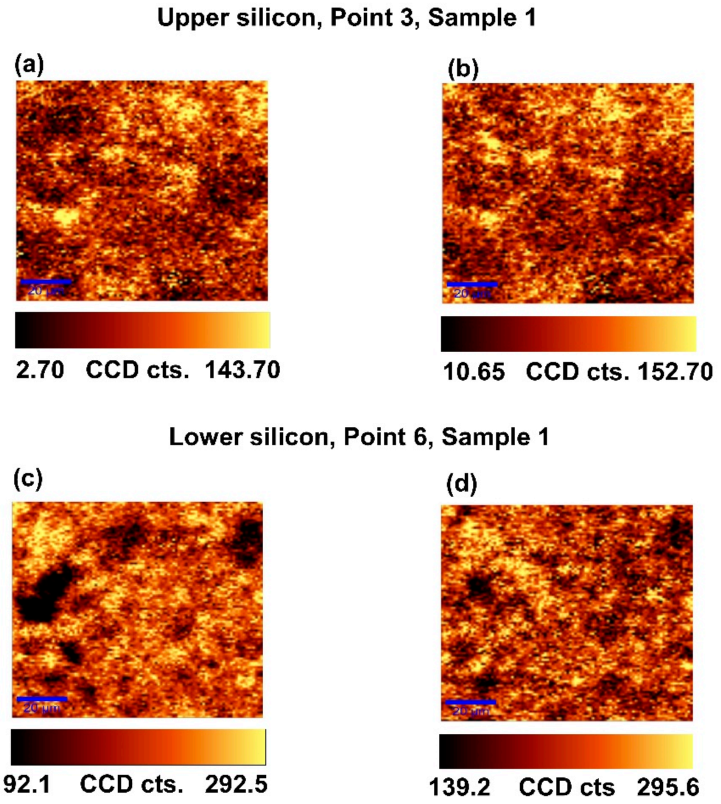

Figure S7. Raman intensity maps of (a, c) the D band and (b, d) the G band for the fracture surface. The fractured EEG paper was positioned at point 3 and point 6 indicated in Figure S6. The mapping area was 100 × 100 µm. Scale bar = 20 µm.
